# Supplementary material for: Synthesis and Hydrophilicity Analysis of bis(propane-1,2-diol) Terminated Polydimethylsiloxanes (PDMSs)
Source: Materials (Basel). 2022 Jan 19;15(3):753. doi: 10.3390/ma15030753 (PMC8836733; doi:10.3390/ma15030753)
Supplement: Supplementary file 1 [file materials-15-00753-s001.zip › materials-1507468-supplementary.pdf]

materials

MDPI

Supplementary Materials

Synthesis and Hydrophilicity Analysis of bis(propane-1,2-diol) Terminated Polydimethylsiloxanes (PDMSs)

Lan-Hee Yang, Kyeong Eun Park and Sungho Yoon *

Department of Chemistry, Chung-Ang University, 84 Heukseok-ro, Dongjak-gu, Seoul 06974, Korea; yangd5d5@cau.ac.kr (L.-H.Y.); pke8597@cau.ac.kr (K.E.P.)
* Correspondence: sunghoyoon@cau.ac.kr

Figure S1 shows the ^{14}C NMR spectra for the structural comparison analysis of the synthesized PDMS (G-PDMS-G-3, G-PDMS-G-2) and glycidol.

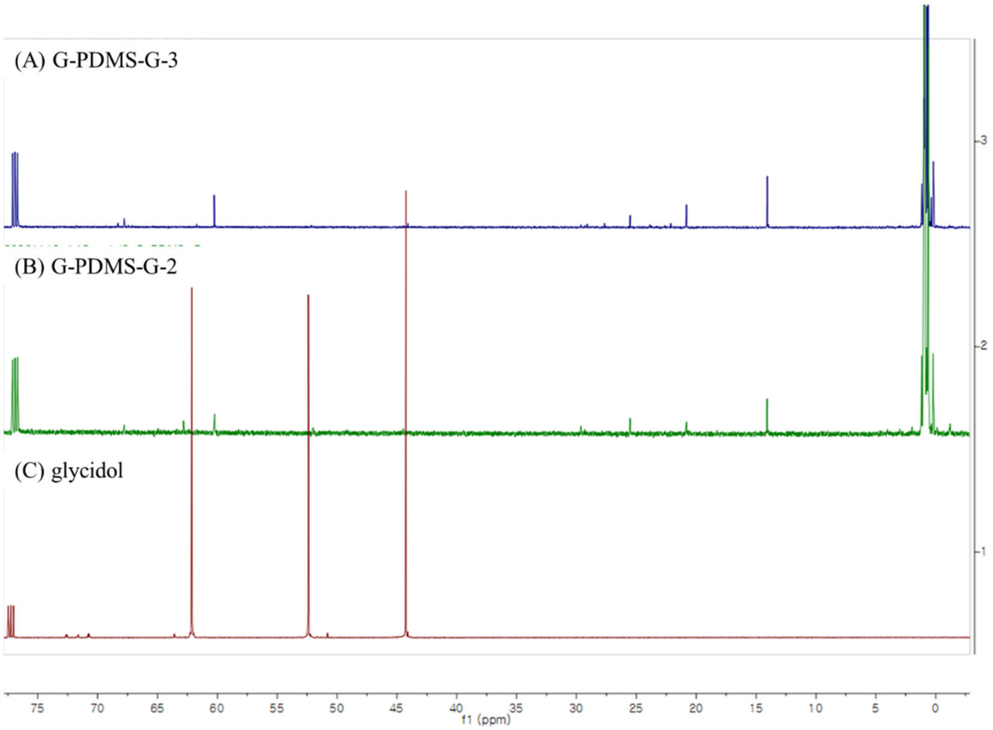

Figure S1. ^{14}C NMR spectra of (A) G-PDMS-G-3, (B) G-PDMS-G-2, and (C) glycidol.

The FT-IR analysis of HO-PDMS-OH-3 and G-PDMS-G-3 is shown in Figure S2. The most important characteristic peaks in the FT-IR spectra are as follows: Si-CH_3 vibration peaks at 1260 and 2950 cm^{-1}, Si-O-Si vibration peaks at 1130-1000 cm^{-1}. In addition, although hydrophilicity can be predicted at the –OH vibration peak of 3200-3550 cm^{-1}, it is difficult to quantitatively analyze the hydrophilicity.

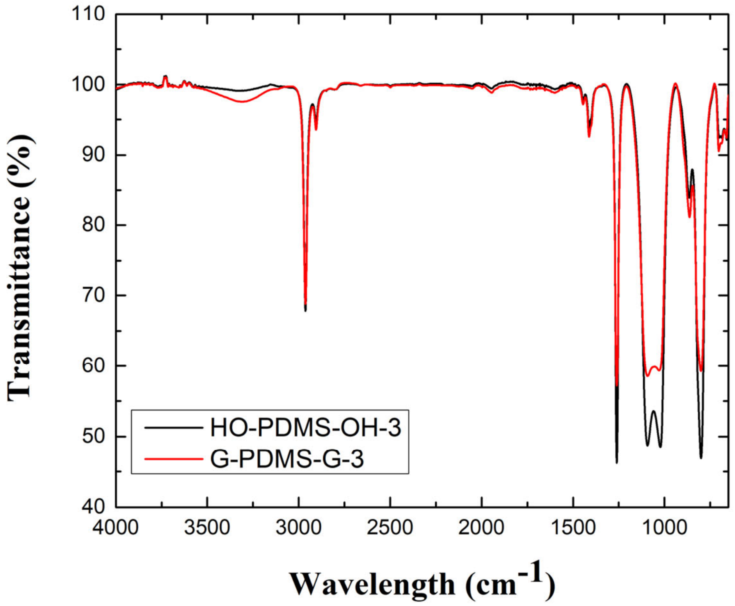

Figure S2. FTIR spectra of HO-PDMS-OH-3 and G-PDMS-G-3.
